# Cyclophosphamide, Thalidomide, and Dexamethasone as Initial Therapy for Patients With Newly Diagnosed Multiple Myeloma in a Middle-Income Country: 7-Year Follow-Up

**DOI:** 10.1200/GO.20.00665

**Published:** 2021-07-23

**Authors:** Jule Vasquez, Rossana Ruiz, Karina Aliaga, Fernando Valencia, Marco Villena, Shirley Quintana, Tatiana Vidaurre, Luis Casanova

**Affiliations:** ^1^ Instituto Nacional de Enfermedades Neoplásicas, Lima, Peru; ^2^ Instituto Oncológico Miraflores, Lima, Peru

## Abstract

Major progress has occurred in multiple myeloma (MM) treatment in recent years, but this is not seen in low- and middle-income countries.

**MATERIALS AND METHODS:**

We retrospectively assessed the efficacy and safety of cyclophosphamide, thalidomide, and dexamethasone (cyclophosphamide 400 mg/m^2^ for 5 days, thalidomide 100 mg once daily, if tolerated, and dexamethasone 40 mg once weekly; in 28-day cycles) in patients with newly diagnosed MM treated at our institution between April 2008 and December 2012. Survival outcomes were estimated by the Kaplan-Meier method.

**RESULTS:**

Fifty-nine patients were found to meet the selection criteria. Median age was 56 years (27-78). Fifty-nine percent (n = 35) were male. International Staging System three was found in 24%. The median number of treatment cycles was 11 (range 4-12). After a median of 81-month follow-up (range 5-138 months), the overall response rate was 69.5%. The complete response and very good partial response were 5% and 32%, respectively. Median progression-free survival (PFS) was 35 months (95% CI, 18 to 41). The 3-year PFS was 47.4% (95% CI, 34.5 to 59.6) and 5-year PFS was 24.9% (95% CI, 14.4 to 36.9). The median of overall survival (OS) was 81 months (95% CI, 33 to not reached). The 3-year OS was 63.4% (95% CI, 49.2 to 74.6), and 5-year OS was 57.5% (95% CI, 43.2 to 69.4). The most common adverse event was neutropenia (grade 3 and 4, 30.5%). Out of 23 patients eligible for stem-cell transplantation, 10 (43.5%) proceeded with autologous transplantation. Treatment-related deaths occurred in four patients (6.7%).

**CONCLUSION:**

Cyclophosphamide, thalidomide, and dexamethasone achieves good response rates with tolerable toxicity, especially in patients age 65 years or younger representing a feasible approach for patients with MM in low-income health care settings.

## INTRODUCTION

Multiple myeloma (MM) is a hematologic malignancy accounting for roughly 10% of all blood cancers.^1^ New cases of MM in United States are more than 30,000 per year.^2^ In Peru, the incidence is estimated at 1.82 × 100,000 for both sexes.^3^ Major changes have happened on the MM treatment from the first case described in 1844,^4^ since steroids, chemotherapy, transplant, proteasome inhibitors, and immunomodulators^5-9^ to newer treatments such as monoclonal antibodies.^10^

**CONTEXT**

**Key Objective**
Multiple myeloma (MM) is one of the hematologic malignancies that has incorporated the largest number of novel treatments as part of its therapeutical arsenal in recent years. However, the benefit of modern therapies is not fully available for countries with limited resources.
**Knowledge Generated**
This study provides new data about long-term outcomes in terms of the efficacy, safety, and survival of patients with MM treated with cyclophosphamide, thalidomide, and dexamethasone as first-line treatment. Patient younger than 65 years are those who have the best benefit with this regimen.
**Relevance**
These findings can be applied to low- and middle-income countries struggling to have access to novel treatments such as bortezomib, lenalidomide, and daratumumab and could be used both for eligible and noneligible patients with MM for autologous transplantation.

In low- and middle-income countries, limited resources in health care make it difficult to have availability of novel drugs. The cost of the myeloma drugs is expensive. Regimens such as bortezomib, thalidomide, and dexamethasone (VTD) or bortezomib, lenalidomide, and dexamethasone (VRD) can cost $100,000 US dollar (USD) or more per year.^11^ In this scenario, a traditional treatment such as cyclophosphamide, thalidomide, and dexamethasone (CTd) is a more affordable alternative for newly diagnosed patients.^12,13^

The aim of this study was to determine the clinical efficacy, safety, and survival outcomes of transplant-eligible and -ineligible patients with MM treated with CTd as first line of treatment in a country with limited resources.

## MATERIALS AND METHODS

### Patients and Staging

We evaluated patients with newly diagnosed MM treated at the National Cancer Institute (Instituto Nacional de Enfermedades Neoplásicas) in Lima, Perú, between April 2008 and December 2012. The diagnosis of MM was based on the WHO 2008 criteria.^14^ Staging was based on International Staging System.^15^ The inclusion criteria were as follows: Diagnosis of MM at Instituto Nacional de Enfermedades Neoplásicas and have received at least four cycles of CTd; for protocol, the evaluation of the treatment response was after 4, 8, and 12 cycles. Exclusion criteria were MM treated previously, patients with another cancer, patients with HIV infection, diagnosis of plasma cell leukemia, and incomplete medical records. This study obtained the written informed consent from all patients and also had the approval of the Institutional Review Board.

During the study period, 181 patients were evaluated, of which 93 patients received CTd, 19 thalidomide dexamethasone, six vincristine, doxorubicin, and dexamethasone (VAD), four cyclophosphamide dexamethasone, five other chemotherapy agents, and seven only radiotherapy; 22 did not meet criteria because of previous treatment; and 25 did not receive any treatment because of loss to follow-up.

In the CTd cohort, 24 received only three or less cycles (25%), and 10 (11%) received four or more cycles, but without assessment of the treatment response or had other exclusion criteria; finally, 59 (64%) patients fulfilled the selection criteria.

### Treatment and Response Criteria

Treatment was based on CTd (cyclophosphamide 400 mg/m^2^ orally for 5 days, thalidomide 100 mg the first week increasing to 200 mg if tolerated and dexamethasone 40 mg weekly, 28-day cycles). All patients received acetylsalicylic acid 100 mg/d as prophylaxis for deep venous thrombosis.^16^ Toxicity was evaluated according to Common Terminology Criteria for Adverse Events version 4.0. Treatment-related mortality (TRM) was defined as death occurring in the absence of progressive cancer and having received CTd in the last 30 days before death.

Response rates were evaluated according to international response criteria after 4, 8, and 12 cycles.^17^

### Statistical Analysis

A descriptive analysis of the information was made through frequencies, percentages, and summary measures. Overall survival (OS) was defined as the time from the date of diagnosis to the date of last control or death. Progression-free survival (PFS) was defined as the time from the date of treatment initiation to the date of last control, progression recurrence, or death. OS and PFS were estimated using the Kaplan-Meier method, and differences were tested with the log-rank test. In the analysis of the information, Stata 15 (StataCorp, TX) was used.

## RESULTS

### Patient Characteristics

The clinical and pathologic characteristics of the patients are shown in Table 1. The median age was 56 years (27-78). The majority were male (59%). In addition, there was a predominance of early stages.

**TABLE 1:**
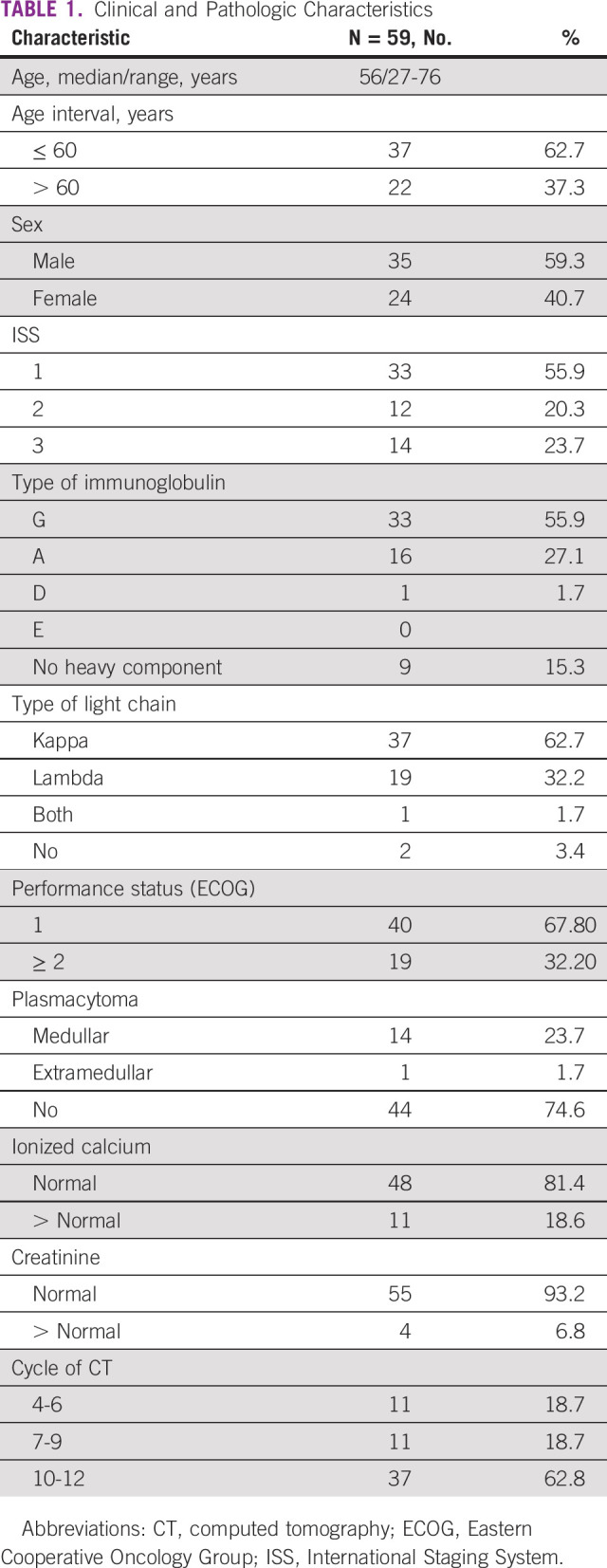
Clinical and Pathologic Characteristics

### Toxicity and Response Treatment

The most common adverse event was neutropenia (44.1%). Neutropenia grades 3 and 4 account for 18.6% and 11.9%, respectively. Also, severe infection was 8.4% and deep vein thrombosis was 6.8%. The toxicities are detailed in Table 2.

**TABLE 2:**
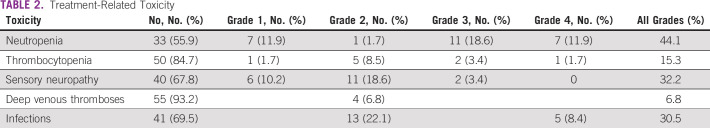
Treatment-Related Toxicity

Regarding the total response rate, it was 69.5%, with a low strict complete response of 1.7%; the other types of responses are detailed in Table 3.

**TABLE 3:**
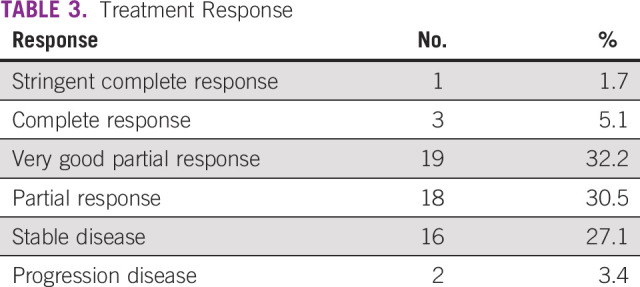
Treatment Response

### PFS and OS

**FIG 1:**
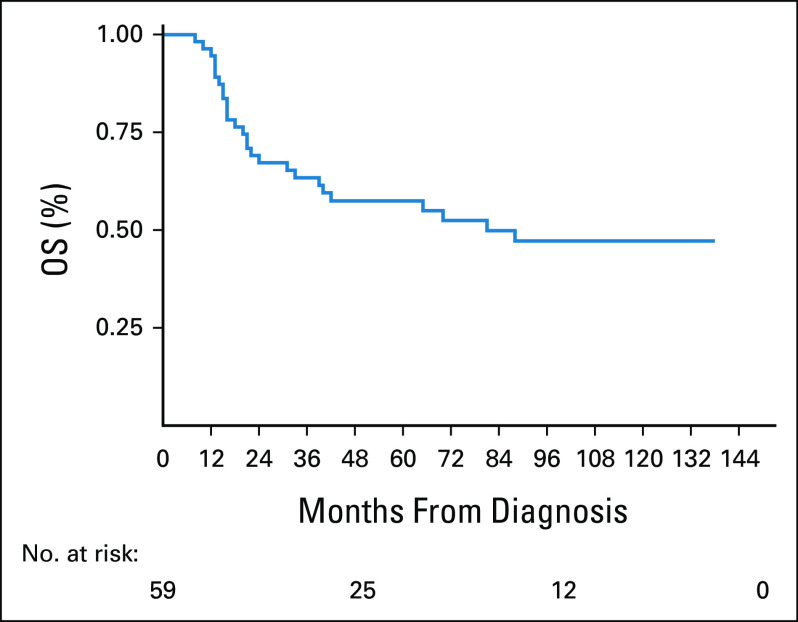
OS of 59 patients with multiple myeloma. OS, overall survival.

The median of follow-up was 41 months (range 5-138 months). The median PFS was 35 months (95% CI, 18 to 41) as shown in Figure 1. The 3-year PFS was 47.4% (95% CI, 34.5 to 59.6), and 5-year PFS was 24.9% (95% CI, 14.4 to 36.9).

**FIG 2:**
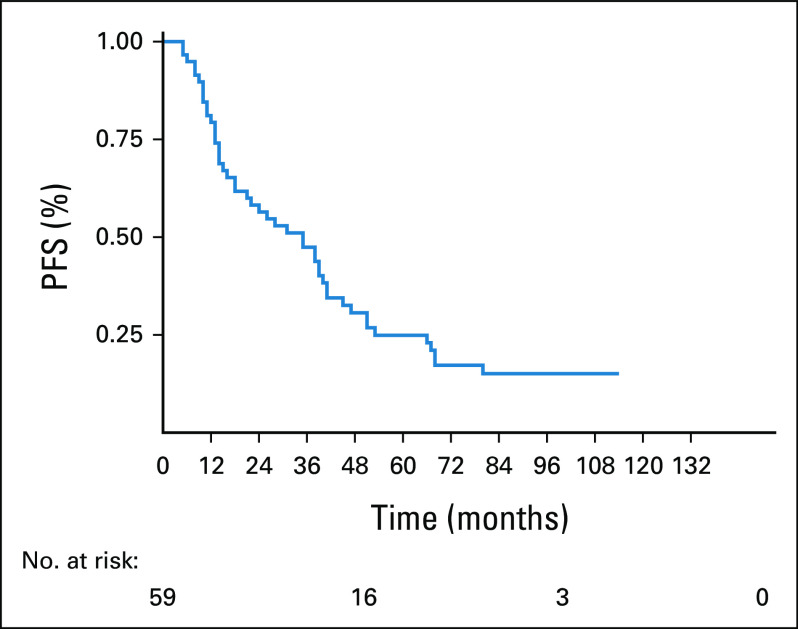
PFS of 59 patients with multiple myeloma. PFS, progression-free survival.

The median of OS was 81 months (95% CI, 33 to not reached). The 3-year OS was 63.4% (95% CI, 49.2 to 74.6), and 5-year OS was 57.5% (95% CI, 43.2 to 69.4) as shown in Figure 2.

### Treatment-Related Mortality

Treatment-related deaths occurred in four patients (6.7%) who were age 62, 64, 74, and 76 years. The median age was 69 years (range 62-76 years), 50% patients were male, 50% achieved at least PR, the median of cycles was 9 (range 6-12), 50% had grade 3 neutropenia, 50% had grade 3 thrombocytopenia, 50% had grade 2 neurotoxicity, three patients died because of pneumonia, and one because of ischemic cerebrovascular disease.

### Transplantation of Hematopoietic Progenitors

The stem-cell transplant program started in our institution in 2012, that is, 4 years after our first patient has received CTd. As an institutional policy, only patients age 65 years and younger were transplanted. Since the late 2011, 33 patients received at least four cycles of CTd, but only 23 patients were eligible for Autologous Stem Cell Transplantation (ASCT) according to the age inclusion criteria. Only 10 (43.5%) patients proceeded with ASCT as a consolidation after induction. The main reason why patients did not proceed onto ASCT was progressive disease (six patients, 46%) followed by limiting comorbidities (three patients, 23%) and failure to collect cells (two patients, 15%). Forty percent (4 patients) of the 10 transplanted patients are on maintenance with thalidomide. The median age was 55 years (range 47-60 years), and 88% were male. The median of treatment cycles was 12 (range, 10-12).

## DISCUSSION

The findings from this study suggest that CTd may be a feasible treatment in a middle-income country such as Peru. The CTd regimen achieves durable responses with tolerable toxicity. The treatment of MM has evolved in our country; until 2006, the most used treatment was VAD.^18^ Thalidomide was available in our institution at the end of 2007 as a salvage treatment. Considering the good outcomes for thalidomide reported in international studies, it was used as part of our front-line treatment since 2008.^19,20^

Studies have shown that thalidomide plus dexamethasone is superior to VAD in terms of response rate and survival.^21,22^ However, these outcomes were modest compared with those reported in a triplet regimen that included cyclophosphamide, such as CTD,^12,13^ and ThaCyDex^23,24^ either in front-line or salvage treatment. The above treatments can be considered not standards in high-income countries where VRD is the first-line treatment in most clinical scenarios. The high cost of this novel regimen, more than $200,000 USD per year, makes it unaffordable in Peru.^11^ Our regimen CTd is an outpatient treatment, but more importantly, a feasible therapy. In Table 4, we can see that the cost of treatment for 1 year of $1,860 USD is almost a sixth cheaper than VTD in Peru, but 60 times than in the United States. Compared to VRD, our treatment is 50 times cheaper in Peru, but 90 times than in the United States. As shown, the administration and the cost of CTd is appropriate for public institutions in Peru.

**TABLE 4:**
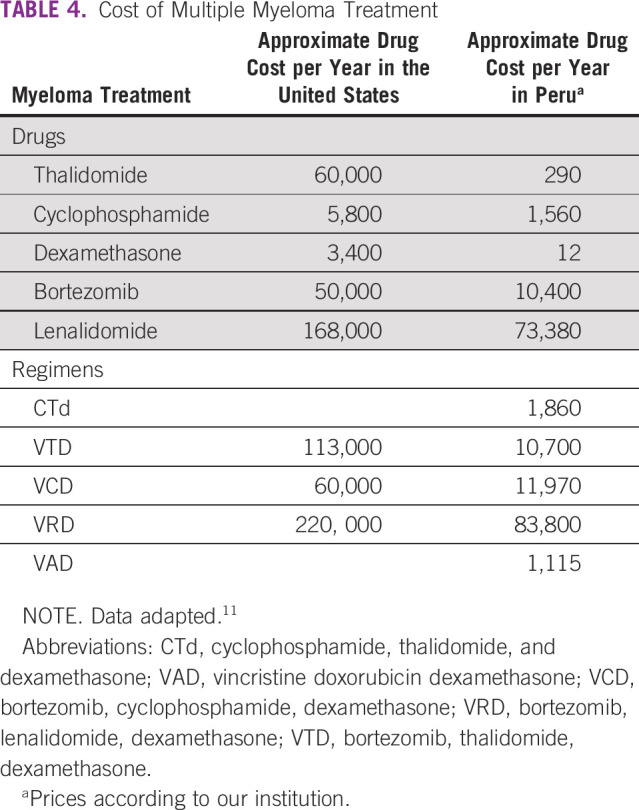
Cost of Multiple Myeloma Treatment

A good regimen also has to be safe and effective. The overall response rate obtained in our study was 69%, which is similar to the 63.8% reported by Morgan's study in patients noneligible for ASCT, however this outcome was worse compared to patients eligible for transplant who had an overall response rate of 82.5%. The treatment regimen in each study was every 21 days and 28 days, respectively. In addition, the dose of cyclophosphamide in both studies was 500 mg weekly. The study presented by Hungria et al^25^ reports even higher overall responses (89.7%). Regarding complete response rates, our study reported 6.8%, which is different from Hungria's study and Morgan's study, which reported 20.7% and 13%, respectively.^13,25^ In Hungria's study, cyclophosphamide was given at 50 mg daily with dexamethasone at high doses (40 mg days 1-4 and 15-18), which is different from the low-dose dexamethasone used in our study (40 mg per week). In our study, cyclophosphamide was given at 400 mg/m^2^ for 5 consecutive days with a total dose higher to the above studies. This different approach tried to improve the intensity of the chemotherapy in the first week instead of weekly as in Morgan's study or in a metronomic way as in Hungria's study. This dose was used uniformly regardless of the eligibility for transplant. The response rates are lower than most recent treatments such as lenalidomide-dexamethasone (RD),^26^ bortezomib, cyclophosphamide, and dexamethasone,^27^ VTD,^28^ and VRD,^29^ which have shown high total response rates that range from 70% to 100%. Thalidomide was given 100 mg daily during the first week, and then was increased to 200 mg; all patients received the full dose of thalidomide since the second week.

The hematologic toxicity in our study was 30% for grades 3 and 4, which is high compared with Morgan's study^13^ for transplant candidates where it was only 4.5% for the same grades; we believe that it could be because of the different way of administering cyclophosphamide in our case: 400 mg/m^2^ × 5 days making a total of 2,000 mg/m^2^, whereas in Morgan's study, 500 mg/weekly making a total of 2,000 mg total dose, meaning an increase of 50% on average. Regarding infections, our work reports 30.5% of infections and Morgan's study reports 20% of grades 3 and 4, which can also be related to the difference in dose. If we focus on peripheral neuropathy, the percentages of grades 3 and 4 are similar in both studies with 3.4% in ours and 3.8% in Morgan's study.

In terms of survival, it is similar with other CTd treatment studies. The PFS in our study was 34 months, which is higher than that in Morgan's study with 27 months,^13^ but similar to those patients who received ASCT in the same study. We believe that a probable cause of this initial difference is that in our work, a more intensive induction regimen was used, but this little advantage is lost when high dose of melphalan is administered as conditioning in transplantation. In our study, the median OS was 81 months with a long-term follow-up (5-138 months). In Morgan's study the median OS was not reached with a shorter follow-up (0-73 months); however, they showed that OS was comparable in both treatment groups, CTd and cyclophosphamide, vincristine, doxorubicin, and dexamethasone, where the latter had a median OS of 63 months^13^ Modern treatments such as RD,^26^ VTD,^28^ and VRD^29^ have OS at 2 years of 87%, 95%, and 97%, respectively, much higher than the nearly 70% in our study (Fig 1). In terms of PFS, RD, VTD, and VRD reported 50%, 50%, and 70%, respectively. By contrast, in our study, the PFS at 2 years was 60%, but with a rapid decrease at 5 years to 25% (Fig 2).

The TRM of our study was 6.7%, discretely below that of Morgan's study^13^ with 8.5%; in addition, among the causes of TRM, infection was observed as the main cause in both studies. We could observe that the deaths of the patients occurred particularly in advanced age (older than 65 years), in females, and in those who received at least six courses of CTd, probably because of low marrow reserve and advanced age. This group of patients may benefit from a 50% reduction in the cyclophosphamide dose.

Patients eligible for ASCT who received transplant in our study were 43.5%, which is different from Morgan's study, which had 67.4% patients eligible. Factors such as progressive disease because of a delay in the preparation process for transplant and lost to follow-up were the main causes of not having a transplant. In our study, the median age was 55 years, similar to that of Morgan's study with 58 years. The failure to collect was 15% in our study, but for the small number of patients, this may be a nonsignificant result, which is different from Morgan's study with 1.96%. In our study only, 40% of patients are on maintenance with thalidomide, which is something that should be corrected.

The main strength of this study is that CTd represents a low-cost regimen, which can be affordable in any low-income country compared with more modern cancer drugs.^30^ In addition, CTd regimen let us avoid the use of outpatient chemotherapy rooms. This oral regimen can be used in patients who are both transplant-eligible and transplant-ineligible. The use of this regimen is not only intended for patients from low-income countries since it is shown that it can be used in combination with novel agents such as carfilzomib, as demonstrated by the CYKLONE study.^31^ We will have to wait for more studies to determine its real value in this era of new agents.

The main limitation of our study is the small number of patients because the cases are from a single institution. Although 181 patients with newly diagnosed MM were admitted in our institution in the study period, only 93 received the standard treatment, 12% were non eligible because of being non-naïve to treatment, and 14% were lost to follow-up. The latter can be the result of both the lack of access to the continuity of cancer care, or that the patients were referred only for diagnosis, considering that our institution is the referral cancer center in Peru. When focusing on the patients who finally received CTd, 25% of the patients were not able to receive at least four cycles. This is mainly because of the discontinuation of treatment for economic issues rather than toxicities as was reported in a Peruvian study, where the abandonment rate was 18%.^32^ Another major limitation of the study is that cytogenetic and fluorescent in situ hybridization studies to categorize patients according to the risk are not available in our institution.

In conclusion, the CTd regimen achieves good response rates with tolerable toxicity, especially in patients age 65 years or younger. In older patients, it is reasonable to reduce full doses. This regimen represents an affordable and effective approach for patients with MM in the scenario of low-income health care with an acceptable toxicity profile.

## References

[1] Kyle RA Rajkumar SV: Multiple myeloma. N Engl J Med 351:1860-1873, 2004 15509819 10.1056/NEJMra041875

[2] Siegel RL Miller KD Jemal A: Cancer statistics, 2016. CA Cancer J Clin 66:7-30, 2016 26742998 10.3322/caac.21332

[3] Payet E; Perez P Poquioma E, et al: Registro de Cáncer de Lima Metropolitana 2004-2005. Estudio de Incidencia y Mortaidad, Volume IV. Lima, Peru, Departamento de Epidemiología y estadística del Cáncer, Instituto Nacional de Enfermedades Neoplásicas, 2013

[4] Solly S: Remarks on the pathology of mollities ossium; with cases. Med Chir Trans 27:435-498.8, 1844 10.1177/095952874402700129 PMC2116942 20895811

[5] Gregory WM Richards MA Malpas JS: Combination chemotherapy versus melphalan and prednisolone in the treatment of multiple myeloma: An overview of published trials. J Clin Oncol 10:334-342, 1992 1531068 10.1200/JCO.1992.10.2.334

[6] Anderson H Scarffe JH Ranson M, et al: VAD chemotherapy as remission induction for multiple myeloma. Br J Cancer 71:326-330, 1995 7841049 10.1038/bjc.1995.65 PMC2033610

[7] Rajkumar SV Blood E Vesole D, et al: Phase III clinical trial of thalidomide plus dexamethasone compared with dexamethasone alone in newly diagnosed multiple myeloma: A clinical trial coordinated by the Eastern Cooperative Oncology Group. J Clin Oncol 24:431-436, 2006 16365178 10.1200/JCO.2005.03.0221

[8] Gay F Hayman SR Lacy MQ, et al: Lenalidomide plus dexamethasone versus thalidomide plus dexamethasone in newly diagnosed multiple myeloma: A comparative analysis of 411 patients. Blood 115:1343-1350, 2010 20008302 10.1182/blood-2009-08-239046 PMC2826759

[9] Cavo M Tacchetti P Patriarca F, et al: Bortezomib with thalidomide plus dexamethasone compared with thalidomide plus dexamethasone as induction therapy before, and consolidation therapy after, double autologous stem-cell transplantation in newly diagnosed multiple myeloma: A randomised phase 3 study. Lancet 376:2075-2085, 2010 21146205 10.1016/S0140-6736(10)61424-9

[10] Palumbo A Chanan-Khan A Weisel K, et al: Daratumumab, bortezomib, and dexamethasone for multiple myeloma. N Engl J Med 375:754-766, 2016 27557302 10.1056/NEJMoa1606038

[11] Rajkumar SV: Value and cost of myeloma therapy. Am Soc Clin Oncol Ed Book 38:662-666, 2018 10.1200/EDBK_200867 30231405

[12] Morgan GJ Davies FE Gregory WM, et al: Cyclophosphamide, thalidomide, and dexamethasone (CTD) as initial therapy for patients with multiple myeloma unsuitable for autologous transplantation. Blood 118:1231-1238, 2011 21652683 10.1182/blood-2011-02-338665 PMC3152492

[13] Morgan GJ Davies FE Gregory WM, et al: Cyclophosphamide, thalidomide, and dexamethasone as induction therapy for newly diagnosed multiple myeloma patients destined for autologous stem-cell transplantation: MRC myeloma IX randomized trial results. Haematologica 97:442-450, 2012 22058209 10.3324/haematol.2011.043372 PMC3291601

[14] Steven H Campo E Harris L, et al: WHO Classification of Tumours of Hematopoietic and Lymphoid Tissues. Multiple Myeloma (ed 4). Lyon, France, International Agency for Research on Cancer (IARC), 2008, pp 200-202

[15] Greipp PR San Miguel J Durie BG, et al: International staging system for multiple myeloma. J Clin Oncol 23:3412-3420, 2005 15809451 10.1200/JCO.2005.04.242

[16] Palumbo A Rajkumar SV Dimopoulos MA, et al: Prevention of thalidomide- and lenalidomide-associated thrombosis in myeloma. Leukemia 22:414-423, 2008 18094721 10.1038/sj.leu.2405062

[17] Durie BG Harousseau JL Miguel JS, et al: International uniform response criteria for multiple myeloma. Leukemia 20:1467-1473, 2006 16855634 10.1038/sj.leu.2404284

[18] Vasquez J Casanova L: Respuesta al Tratamiento de Primera Línea de los Pacientes con Mieloma Múltiple, Instituto Nacional De Enfermedades Neoplásicas 2002-2006. Revista Acta Cancerológica, Volumen 42, Enero-Junio 2013, pp 7-16

[19] Richardson P Anderson K: Thalidomide and dexamethasone: A new standard of care for initial therapy in multiple myeloma. J Clin Oncol 24:334-336, 2006 16365174 10.1200/JCO.2005.03.8851

[20] Rajkumar SV Hayman S Gertz MA, et al: Combination therapy with thalidomide plus dexamethasone for newly diagnosed myeloma. J Clin Oncol 20:4319-4323, 2002 12409330 10.1200/JCO.2002.02.116

[21] Cavo M Zamagni E Tosi P, et al: Superiority of thalidomide and dexamethasone over vincristine-doxorubicindexamethasone (VAD) as primary therapy in preparation for autologous transplantation for multiple myeloma. Blood 106:35-39, 2005 15761019 10.1182/blood-2005-02-0522

[22] Vogl DT Liu SV Chong EA, et al: Post-transplant outcomes of induction therapy for myeloma: Thalidomide and dexamethasone versus doxorubicin, vincristine, and dexamethasone prior to high-dose melphalan with autologous stem cell support. Am J Hematol 82:1071-1075, 2007 17696204 10.1002/ajh.21038

[23] Garcia-Sanz R Gonzalez-Fraile MI Sierra M, et al: The combination of thalidomide, cyclophosphamide and dexamethasone (ThaCyDex) is feasible and can be an option for relapsed/refractory multiple myeloma. Hematol J 3:43-48, 2002 11960395 10.1038/sj.thj.6200150

[24] Garcia-Sanz R Gonzalez-Porras JR Hernandez JM, et al: The oral combination of thalidomide, cyclophosphamide and dexamethasone (ThaCyDex) is effective in relapsed/refractory multiple myeloma. Leukemia 18:856-863, 2004 14973508 10.1038/sj.leu.2403322

[25] Hungria VT Crusoe EQ Maiolino A, et al: Phase 3 trial of three thalidomide-containing regimens in patients with newly diagnosed multiple myeloma not transplant-eligible. Ann Hematol 95:271-278, 2016 26518211 10.1007/s00277-015-2537-2

[26] Rajkumar SV Jacobus S Callander NS, et al: Lenalidomide plus high-dose dexamethasone versus lenalidomide plus low-dose dexamethasone as initial therapy for newly diagnosed multiple myeloma: An open-label randomised controlled trial. Lancet Oncol 11:29-37, 2010 19853510 10.1016/S1470-2045(09)70284-0 PMC3042271

[27] Reeder CB Reece DE Kukreti V, et al: Cyclophosphamide, bortezomib and dexamethasone induction for newly diagnosed multiple myeloma: High response rates in a phase II clinical trial. Leukemia 23:1337-1341, 2009 19225538 10.1038/leu.2009.26 PMC2711213

[28] Moreau P Avet-Loiseau H Facon T, et al: Bortezomib plus dexamethasone versus reduced-dose bortezomib, thalidomide plus dexamethasone as induction treatment before autologous stem cell transplantation in newly diagnosed multiple myeloma. Blood 118:5752-5758, 2011; quiz 982 21849487 10.1182/blood-2011-05-355081

[29] Richardson PG Weller E Lonial S, et al: Lenalidomide, bortezomib, and dexamethasone combination therapy in patients with newly diagnosed multiple myeloma. Blood 116:679-686, 2010 20385792 10.1182/blood-2010-02-268862 PMC3324254

[30] Aguiar PM Lima TM Storpirtis S: Systematic review of the economic evaluations of novel therapeutic agents in multiple myeloma: What is the reporting quality? J Clin Pharm Ther 41:189-197, 2016 27009796 10.1111/jcpt.12384

[31] Mikhael JR Reeder CB Libby EN, et al: Phase Ib/II trial of CYKLONE (cyclophosphamide, carfilzomib, thalidomide and dexamethasone) for newly diagnosed myeloma. Br J Haematol 169:219-227, 2015 25683772 10.1111/bjh.13296 PMC4521972

[32] Vasquez L Diaz R Chavez S, et al: Factors associated with abandonment of therapy by children diagnosed with solid tumors in Peru. Pediatr Blood Cancer 65:e27007, 2018 29431252 10.1002/pbc.27007

